# “The package has been opened”- parents' perspective and social validity of an Early Start Denver Model intervention for young children with autism

**DOI:** 10.3389/frcha.2024.1509828

**Published:** 2024-12-04

**Authors:** Emilia Carlsson, Gudrun Nygren, Christopher Gillberg, Petra Linnsand

**Affiliations:** ^1^ Gillberg Neuropsychiatry Centre, Institute of Neuroscience and Physiology, University of Gothenburg, Gothenburg, Sweden; ^2^ Research Department, Angered Hospital, SV Hospital Group, Gothenburg, Sweden; ^3^ Speech and Language Pathology Unit, Institute of Neuroscience and Physiology, University of Gothenburg, Gothenburg, Sweden; ^4^ Child and Adolescent Specialist Centre, Angered Hospital, SV Hospital Group, Gothenburg, Sweden

**Keywords:** early intervention, social validity, autism, preschool children, parents' views

## Abstract

**Introduction:**

This study aimed to capture experiences and perspectives of parents of children with autism participating in an intervention program based on the Early Start Denver Model (ESDM). Specifically, we wanted their views regarding feasibility, acceptability, and significance of the intervention program, i.e., its social validity.

**Methods:**

Fourteen parents, whose children has been diagnosed with autism, were interviewed.

**Results:**

The results included three themes (1) *Comprehensive approach*: the participants emphasized the importance of early detection and interventions in their local setting in close cooperation between themselves, health care professionals, and preschool staff. They also highlighted the individual goals based on the child's needs in different developmental areas, as well as the whole family's needs and prioritizations. (2) *Hands on—available locally and accessible:* focused on different aspects of procedures, including features of the ESDM, parent education, the parent-therapist relationship and nearby location. (3) *Sense of empowerment—parents got increased knowledge:* the intervention was significant within family daily living and daily activities. The participants expressed that the interventions program contributed to an increased knowledge about autism and the ESDM strategies, positively impacted their child, and improved the collaboration with the preschool.

**Conclusion:**

Parents emphasized the naturalistic, comprehensive, and local setting of the intervention and described that they had gained new knowledge as well a sense of empowerment. The results indicated that the intervention program based on the ESDM was socially valid according to parent descriptions.

## 1 Introduction

Autism is a neurodevelopmental disorder (NDD) characterized by early childhood onset and typically following a lifelong course (1). It is estimated that approximately 1% of children worldwide receive an autism diagnosis, but prevalence rates vary considerably (2). Also, studies have reported a higher prevalence of autism in migrant populations (3–8).

Early interventions for young children with autism are considered important, and Dawson (9) has underscored the second year of life as a crucial period characterized by significant neural plasticity affording a greater potential to influence developmental course. Early interventions have been shown to enhance developmental outcomes (10, 11). The Early Start Denver Model (ESDM) is one of the most well-established intervention models for young children with autism, demonstrating promising results, including improvement in core developmental domains and reduction of maladaptive behaviors (12, 13).

The ESDM is a Naturalistic Developmental Behavioral Interventions (NDBI) and integrates principles of developmental science, applied behaviour analysis, and social-affective neuroscience to promote learning and development in young children with autism (10, 14). ESDM's essential features are the focus on developmental-based learning targets and elementary social learning skills, for example joint attention and imitation. The learning context includes the child's daily interactions, experiences, and play routines within the natural environments. The intervention is highly child-directed and use techniques to stimulate spontaneity, initiative, and generalized skills, including incorporating family members and other caregivers in the interventions. The use of ESDM has been established for very young children with autism (aged 12–48 months) focusing on autism-specific impairments spanning various developmental domains, including expressive and receptive language, social interaction, and play skills (15).

The ESDM is designed to be implemented across different settings and delivery formats, including therapist-delivered, parent-mediated, and teacher-delivered group-based program in preschool settings. In the parent-implemented ESDM (P-ESDM), parents are coached to deliver the intervention in the child's daily activities (16). The parents undergo training in basic ESDM strategies, thus facilitating implementation across daily activities built on the child's spontaneous interest and motivation. Several studies have demonstrated that such training is effective in improving parent use of ESDM techniques, and that child outcomes may be improved in one or more domains (17–19). Another study of P-ESDM has shown positive outcomes on parents but not regarding the child's performance (20).

Vivanti and Stahmer (21) highlighted the importance of understanding how parents perceive different approaches to the delivery of an early intervention. This is more evident in parent-delivered and low-intensity therapies which are among the most cost-effective and accessible methods of delivering early interventions. This is especially important when adapting interventions to marginalized populations, such as ethnic minorities and people living in low-resource settings. When implementing interventions in natural and authentic environments, social validity is a pivotal concept. Wolf (22) has conceptualized three different aspects of social validity including *goals of the intervention* (i.e., if the intervention target is socially significant for the participants), *acceptability of procedures* (i.e., if the intervention procedures are acceptable and feasible for achieving the goal), and *social importance of intervention outcomes* (i.e., if the intervention is effective and the outcomes are socially significant). Social validity may impact the implementation of the intervention in natural settings (23). Therefore, it is essential to evaluate social validity so as to increase the likelihood of an intervention being implemented and maintained. Horner et al. (24) have presented criteria that can increase the social validity of the: intervention (a) evidence that intervention agents, e.g., parents, can implement the intervention with fidelity in natural settings; (b) reports from agents that the intervention procedures are acceptable, feasible within available resources, and effective; and (c) evidence that the interventions implementation continues even after support has ended.

Several studies have demonstrated the social validity of the ESDM across indicators of acceptability, satisfaction with the intervention, and collaboration between professionals and caregivers (17, 18, 25, 26). In a study by Ogilvie and McCrudden (27), parent perceptions of the social validity of the ESDM were evaluated. They found that parents perceived that the ESDM had social validity, had satisfactory procedures, and produced meaningful results. Rogers et al. (20) studied the feasibility of parent-implemented ESDM in low-resource communities. The provider-parent fidelity was good, and both providers and parents demonstrated significant improvements on the fidelity of implementation scores with moderate effect sizes compared to control groups. However, no differences were observed between the groups in child developmental scores. Also, van Noorden et al. (28) found that parents experienced a P-ESDM program that included both group and individual delivery P-ESDM as feasible, accessible and effective. Strong therapeutic alliances, individualized goals, and a safe learning environment were described as factors that impacted the accessibility of the program.

Despite the potential benefits, opportunities for interventions for children with autism are often lacking, particularly to people with migrant background (8, 29). For instance, Amant et al. (29) have found that children with autism whose parents did not speak the country's main language as their primary language received significantly fewer hours of services provided by their state disability program. Sritharan and Koola (30) underscored that culturally sensitive program may address barriers and provide equal healthcare service for families and children with migrant backgrounds.

Along these lines, our multidisciplinary team working in an area in the city of Gothenburg, Sweden, with very high frequency of people with migrant backgrounds and/or having low socioeconomic status, have developed a local assessment of children with NDDs, (particularly autism) and low-intensity intervention program based on the ESDM.

This study aimed to qualitatively capture perspectives of parents of children with autism participating in the interventions program based on the ESDM. Specifically, we studied their views regarding the social validity of the intervention program.

## 2 Materials and methods

### 2.1 Study design

To be able to capture the lived experience and in-depth knowledge from the included parents, a qualitative method was chosen. Qualitative in-depth interviews were conducted and analyzed by using content analysis in two steps, both inductive and deductive approaches (31).

### 2.2 Study context

The study was performed in an area in Gothenburg in Sweden, where the vast majority of the population is either born outside Sweden, of have two parents who were (32). The area has a high prevalence of ill health, a high unemployment rate, and a low average income. In an earlier study from this area, we have reported a high prevalence of autism, 3.7%, in preschool children (33).

A multidisciplinary team provides assessment and interventions for young children affected by NDDs, particularly autism, close to where the families live, and in cooperation with the healthcare nurse at the local Child Health Centre (CHC), the preschool staff, and other social services. The multidisciplinary team's primary objectives are to facilitate assessment and intervention for these families locally, thereby enhancing the accessibility and continuity of healthcare services while also encouraging active parental participation in the interventions. For more information about the multidisciplinary team, see Linnsand et al. (33). The intervention program for children with autism was a low-intensity program based on the ESDM, see the section Procedure in Methods.

In Sweden, preschool is not compulsory, but approximately 85% of children aged 1–5 years do attend preschool. Preschool education is the first step in the Swedish education system and it is incorporated in the Swedish Education Act (34). Nearly all children with special educational needs in Sweden are enrolled in regular preschool. For more details about the Swedish preschool, see Linnsand et al. (35).

### 2.3 Participants

The study participants were parents whose children had been diagnosed with autism by the multidisciplinary team.

Fourteen parents of eleven children were interviewed. The age of the parents at the time of intervention ranged from 24 to 49 years (mean age 37). None of the parents had Swedish as their native language. Somali and Arabic were the most common native language. The parents’ years of schooling were as follows: five had a vocational education, three had completed upper secondary school, and four had completed compulsory school. Data was missing for two participants.

The eleven included children had been diagnosed with autism between 1 and 3 years of age. In addition to the autism diagnosis, all children met criteria for at least one neurodevelopmental disorder, including sleeping- and eating problems, early ADHD symptoms, speech and language problems, learning disabilities and/or intellectual developmental disorder. From the second year of life, all included children attended preschool. All children received the intervention based on the ESDM, and the intervention period ranged from 1 to 2 years. Participant demographics are summarized in Table 1.

**Table 1:** Demographic description of participating parents (*n* = *14*) and their children.

Parent (no.)	Age of parent (years)	Sex	Education	Age of child at diagnosis (years)	Severity level of autism	Intervention period (years)	Interpreter
1.	34	F	Vocational education	3 3	Level 1 Level 1	2 2	No
2.	24	F	Upper secondary school	3	Level 2–3	1.5	No
3.	34	F	Vocational education	2	Level 2	2	Yes
4.	24	F	Vocational education	2	Level 2	1.5	No
5.	33	M	Compulsory school	2	Level 2	2	No
6.	44	M	Compulsory school	3	Level 1–2	2	Yes
7.	33	F	Compulsory school	2	Level 2	2	Yes
8.	43	M	Compulsory school	2	Level 2	2	Yes
9.	42	F	Data missing	1 3	Level 2 Level 1	1 1	Yes
10.	27	F	Upper secondary school	2	Level 1	2	No
11.	29	F	Vocational education	2	Level 1	2	No
12.	32	F	Vocational education	1	Level 1–2	2	No
13.	36	M	Upper secondary school	2	Level 1	2	No
14.	49	M	Data missing	1 3	Level 2 Level 1	1 1	Yes

Three of the included parents had two included children.

### 2.4 Recruitment

In the recruitment, a purposive sample strategy was applied, specifically targeting participants that were aligned with the study's aim. They were recruited with the aim of capturing as broad a range of experiences as possible, with respect to age, education, language background, and sex. The participants should be parents of at least one child with autism and have participated in the intervention program based on the ESDM for at least one year.

To be able to shed light on the research questions from as many different potential views as possible, the sample included mothers and fathers with one or two children with autism, children with autism and different comorbidity difficulties, and parents who needed an interpreter or not.

Fifteen parents met the inclusion criteria's and were invited to participate in the study by a member of the multidisciplinary team. Fourteen parents expressed interest in participation and were contacted by the author (PL), who gave verbal information about the study. Parents who spoke fluent Swedish were contacted by telephone, and they received a description about the aim of the study and procedure. Parents who did not speak fluent Swedish were informed about the study during a visit to the multidisciplinary team, with the help of an interpreter. Fourteen parents consented to participate, they received written information about the study and signed an informed consent form. They were also informed about their right to end their participation at any time without negative consequences for their child's continued treatment.

### 2.5 Data collection

Thirteen interviews were conducted; one included both mother and father, while eight interviews included mothers and four fathers. In two families, the mother and father participated in different interviews. The interviews were conducted during November and December 2022.

The interviews took place at the local child-health unit and followed a semi-structured interview guide, creating opportunities to pose follow-up questions and, in that way, to be able to capture the lived experiences of the parents. The interview guide was developed by three of the authors (EC, PL, and GN). PL (clinical psychologist/preschool teacher) and GN (pediatrician/child and adolescent psychiatrist) are members of the multidisciplinary team. PL and GN are certified ESDM therapists and have developed the intervention program based on ESDM. EC (speech-language pathologist), who was not a part of the multidisciplinary team but a part of the study due to in-depth knowledge in qualitative methods and in conducting interview studies.

The interview guide was developed based on a literature review by conceptualizing the aim of the study and using clinical knowledge. The guide included open-ended questions, such as, “*Tell me about the work of the intervention program in collaboration with the preschool and the healthcare service*”. “*How have you worked with the strategies in everyday life?*” and “*What opportunities and obstacles have you experienced in working with the intervention program?*”.

The interviews were conducted and audio-recorded by the author (EC). The interviews lasted 20–60 min and were transcribed verbatim by the author (PL). Six interviews were performed with an authorized interpreter, for the other interviews no interpreter was needed. When uncertainties arose, the interviewer asked for clarification. To avoid potential language bias clarifications was used more frequently to ensure that the parents conveyed their message clearly. Data saturation was estimated, by discussions between the authors, to have been reached after the 13th interview, i.e., the point in the research process where no new and relevant information to the aim emerged.

### 2.6 Data analysis

The interviews were analyzed in two steps, first by inductive content analysis (31), by authors PL and EC. Meaning units were identified, condensed, and coded, uncertain codes were discussed between PL and EC to confirm methodological rigor and trustworthiness of the results (36, 37). Content that shared commonality was categorized and reflected upon, latent content was interpreted through discussions where subthemes and themes were discussed between the authors (38). See Table 2 for examples of the analysis.

**Table 2:** Examples from the analytic process, the content analysis—from quote to theme.

	Theme	Subtheme	Code	Quote
*Goals of the intervention program*	Significance of the intervention's goals: A comprehensive approach	Early detection—early intervention	That the child got support early is important	“*You don’t want to miss anything, you want to make the most of your time. It is a limited time*”. (4)
		Collaboration between parents, healthcare and preschool	A multi-professional team—a broad intervention that covers a lot and the collaboration between profession is essential	“*I think it is good that they [preschool staff] get training by attending the visit with the team not just through paper works*”. (11)

Findings were analyzed further by sorting themes and subthemes based on social validity framework developed by Wolf (22). When disagreements regarding interpretation arose, the authors engaged in thorough discussions to reach a consensus. The software NVivo 12 was used to structure data (39). To ensure anonymity, details such as gender, and names were removed from the transcript and participants were coded to a specific number when quotes appeared within the text to safeguard their identity.

### 2.7 Procedure

The main goal of the ESDM is to promote the child's development in multiple domains of communication, social and play skills, cognitive abilities, adaptive functions, gross- and fine motor skills (15).

In this study, the intervention program was a low-intensity program based on the ESDM. Parents and preschool staff used the ESDM strategies in the child's natural setting for 12 to 24 months. A certified ESDM therapist or a trained ESDM parent-coach coached the parents and the preschool staff, assisting them in implementing the program's objectives and ESDM strategies into everyday caregiving, play activities, and routines at home and in preschool (16).

Some adjustments were made to the program to adapt it to the needs of families with migrant background, including simplifying the objectives and learning steps. The ESDM therapist coached the parents through the steps. If required, the therapist visited the family's home to apply the objectives and ESDM strategies in day-to-day life. Also, simplification of language was needed, as some of the parents could not read Swedish or were illiterate. Interpreters were used in more than 60% of the visits. However, all fundamental elements of the ESDM methodology were maintained.

### 2.8 The intervention program based on the ESDM

#### 2.8.1 Step 1. Group-based education for parents and preschool teachers

**Figure 1:**
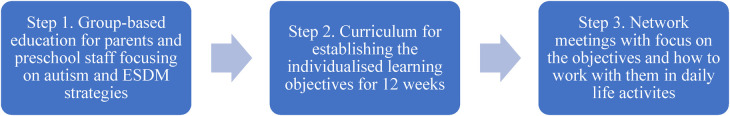
The intervention program based on the ESDM and the different steps (step 1–3).

The parents and preschool staff participated in an education program focused on autism and ESDM strategies. It included knowledge of autism signs, underlying factors contributing to autism, and co-existing NDDs. Furthermore, it incorporated information on ESDM and strategies for creating opportunities for communication and interaction within daily activities. The parental education lasted 3 h on two different occurrences. The education for the preschool staff was a group-based digital interactive presentation of a single section of 3.5 h (Figure 1).

#### 2.8.2 Step 2. Curriculum checklist for establishing individual learning objectives in young children with autism

In the first session, the therapist and parents played with the child to find developmentally appropriate goals on the ESDM curriculum checklist. That curriculum was used to broadly evaluate the child's capabilities across numerous developmental domains (receptive communication, expressive communication, imitation, play, joint attention, social skills, cognition, fine motor, gross motor, behaviour, and personal independence) and to create individualized teaching objectives. In addition, based on interviews with the parents and the preschool curriculum, the ESDM therapist formulated objectives for each child. The objectives were also based on the parents' learning profile and identified priorities, i.e., goals considered crucial to work on within the home setting (40).

The curriculum was administrated every 12 weeks, and new objectives were constructed when the previous had been fulfilled. An objective was accomplished when the child reached the specific ability/task across activities and settings.

#### 2.8.3 Step 3. Network meetings

A network meeting was assembled every second week, including the child, the parents, the preschool staff, and the ESDM therapist. These meetings mostly took place at the clinic, but sometimes at home or preschool of the child. Then, the therapist coached the parents and the preschool staff in applying the child's individualized objectives in everyday activities. The meetings also included a discussion of progress from the previous weeks and goal setting for the upcoming two weeks.

The parents, preschool staff, and the ESDM therapist jointly determined the focus of the meetings. The meetings followed a structure that included a greeting ritual, playtime, song and rhymes, a break for snacks, book reading, and a goodbye ritual. During the network meetings, the therapist explained and demonstrated the ESDM techniques, such as child-initiated teaching episodes, modelling, and using natural reinforcement. The therapist provided opportunities for parents and the preschool staff to practice strategies and interact with the child, offering constructive feedback and support. Sometimes, the parents showed how they used the strategies at home, and the preschool staff could employ the same strategies in preschool and vice versa. The parents and preschool staff implemented the objectives in the child's day-to-day life between the meetings. For more information about the intervention program implemented in the preschool setting, see Linnsand et al. (35).

## 3 Results

The study's aim was to describe how parents to children with autism experienced participating in the intervention program based on ESDM. Specifically, the study was aimed at compiling the parents' views regarding the social validity of the intervention program: (1) s*ignificance of intervention goals* (2) a*cceptability of procedures*, and (3) *social importance of intervention outcomes*. The analysis revealed three themes, (1) *Comprehensive approach*, (2) *Hands on—available local and accessible* and (3) *Sense of empowerment—parents got increased knowledge.* An overview of the results is presented in Table 3.

**Table 3:** Results presented in themes, subthemes and overarching theoretical framework.

	Significance of the intervention's goals	Acceptability of procedures	Social importance of intervention outcomes
Theme	Comprehensive approach	Hands on—available locally and accessible	Sense of empowerment
Subtheme	Early detection—early intervention	ESDM features—a naturalistic approach	Increased knowledge—a sense of comfort and control
	Boosting the child's development in multiple areas	To gain new knowledge—parent education	Positive impact but still a need for intervention
	Individual goals and strategies in daily activities based on the family's need and prioritization	Essential parent-therapist relationship	Improved collaboration
	Collaboration between parents, healthcare and preschool	To operate locally—increased accessibility	

### 3.1 Significance of the intervention's goals: comprehensive approach

The goals of the intervention were described as comprehensive, for instance, the participants emphasized the importance of early detection and interventions in their local setting in close cooperation between themselves, healthcare professionals, and preschool staff. They also highlighted the individual goals based on the child's needs in different developmental areas, as well as the whole family's needs and prioritizations. The participants described the goal of the intervention program as socially significant.

#### 3.1.1 Early detection—early intervention

The parents emphasized the significance of early support when the child had a deviant development. Some of them were afraid of missing the opportunities for early interventions that could reduce symptoms of autism. “*You don’t want to miss anything; you want to make the most of your time. It is a limited time*” (4). Even before the assessment, the parents needed advice on how to interact with their child. Some of them described a lack of knowledge and experience of children with difficulties as well as typically developing children. “*It is our first child. We had no experience. So, if we were to know which activities to do with the child. We learned it from the CHC*” (7).

The parents described how the multidisciplinary team facilitated early assessment and intervention. “*The healthcare system doesn’t have enough time. I am so fortunate to have gotten a spot in this program. I don’t want my son to get worse. I started with him immediately*” (2).

#### 3.1.2 Boosting the child's development in multiple areas

The child required support within the core domains of autism, including social communication and interaction, lack of eye contact, poor vocabulary, and difficulties playing with other children. “*She still has communication difficulties. She doesn’t speak*” (4). Additionally*,* the parents describe that the child had a need for support within different domains, including sleeping, eating, and basic needs such as toilet training. Some of the parents also expressed that their child had behavioural problems and a high activity level, which led to a constant need for supervision by the parents”. *She is very quick and active, so you always must be with her. You can't leave her alone*” (11). The parents said that the intervention addressed difficulties in different developmental areas contributed to the significance of the intervention.

### 3.2 Individual goals and strategies in daily activities based on the family's needs and prioritizations

The parents expressed diverse needs, both concerning the child but also themselves. Having a child with difficulties means navigating and maintaining regular contact with different professionals, such as in healthcare, social services, and preschool. They described challenges in understanding and navigating the healthcare system and other service systems, such as the Swedish Social Insurance Agency. In that way, they needed support to establish contact with different social services units. “*We have received so much support. An unusually large amount. /…/ They want things to work out for us*” (13).

#### 3.2.1 Collaboration between parents, healthcare and preschool

The parents described that the collaboration between them, healthcare professionals, and preschool staff was important. They reported that healthcare staff had an essential role as providers of knowledge about the child's development and needs to the preschool staff, but also to convey more general knowledge about autism and how the preschool staff could help the child in everyday life. “*I think it is good that they [preschool staff] get training by attending the visit with the team, not just through paperwork*” (11). According to the parents, the ESDM therapist also had an essential role as mediator between the parents and preschool staff. The preschool staff participated in the clinic's intervention sessions, making the ESDM strategies transferable across the two settings.

### 3.3 Acceptability of procedures: hands on, locally available and easily accessible

Acceptability of the procedures in the intervention was identified by the second theme, the intervention was described by the parents as being hands on, available and accessible. The theme comprised four subthemes, focused on different aspects of procedures, including features of the ESDM, parent education, and the importance of the parent-therapist relationship and the multidisciplinary team´s local location.

#### 3.3.1 ESDM features—a naturalistic approach

Parents emphasized the significance of the naturalistic approach in the intervention, i.e., using daily activities to create learning opportunities, contributed to the procedures' acceptability. A central aspect of the ESDM was “let the child lead”, and the parents experienced that the focus on the child's interest and motivation made it possible to integrate the intervention in their daily life.

Parents highlighted the shared objective-setting and network meetings as vital elements of the intervention program based on the ESDM. During the network meeting, the parents experienced the value of receiving coaching. The parents described the individualized goals as valuable. The goals became significant when they were based on the child's need, as well as the whole family's needs. “*Goals have been set at every meeting, they have based the goals on us, both the therapist and preschool [staff]*” (12). Also, the parents said that it was essential that the goals were applicable to their daily routines and could be integrated into the family's daily life.

#### 3.3.2 To gain new knowledge—parent education

The content of the parental education given by the professionals weas perceived as useful and important. The parents expressed that they received essential knowledge about autism and strategies for interacting with their children. “*I received parental education, so I got a lot of information about autism. How we as parents can learn to communicate with our child*” (1).

Specific skills and communication strategies were taught by the ESDM therapist and practiced during the meetings at the clinic. The strategies and skills were valuable and valid for use within the family's daily life. “*To guide her. /…/ She [therapist]) told us that we shouldn’t talk using too many words to our girl*” (9).

Some parents wanted to inform older siblings or other relatives about autism and how they could support the child with autism. “*X [therapist]had a home visit at our house, a meeting with the whole family. Older siblings talked about how they could help NN [the child with autism]. That meeting helped us to have a common [improved] understanding of how we could help [the child with autism]*” (6).

#### 3.3.3 Essential parent-therapist relationship

Parents experienced that the relationship with the ESDM therapist had been of great importance. They felt that they were able to contact the therapist regarding their child but also other things such as contacts with authorities. The availability and helpful attitude of the therapist were essential for the parents. They talked about the importance of the therapist's availability for emotional support. “*You don’t always feel well, so sad, or something. I used to contact. Specially phone. If the phone didn’t work, I came straight away*” (11).

#### 3.3.4 To operate locally—increased accessibility

To travel a long distance with a small child with large social difficulties could be a challenge according to the parents' descriptions. Therefore, the parents described the importance of the locally based team, it was perceived as accessible and highly acceptable. The parents experienced comfort and security that were valuable to them. They felt it was easy to contact the ESDM therapist. “*Here is a lot of security. In everything. Here is the health centre, the rehabilitation centre and the preschool. I think it is very good*” (1).

### 3.4 Social importance of intervention outcomes: a sense of empowerment

The social importance of the intervention outcomes was identified as a sense of increased empowerment. The parents described that the intervention was significant within the family's daily living and their activities. The parents expressed that the intervention program contributed to an increased knowledge about autism and the ESDM strategies positively impacted their child and improved the collaboration with the preschool.

#### 3.4.1 Increased knowledge—a sense of comfort and control

This subtheme's content included increased knowledge about autism and ESDM strategies, which contributed to a sense of empowerment and hope for the future among the parents.

The word autism was experienced as a totally new concept for several parents, and they needed knowledge to understand and support the child. The parents pointed out that both knowledge of autism and ESDM strategies were important. “*I had no knowledge about autism/…/when I lived in Somalia, no one talked about autism*” (13). The parents described that the enhanced knowledge about autism contributed to a sense of security and control, then they recognized how they could support the child. “*Then I felt safe. That's security*” (12). The parents also highlighted the importance of increased knowledge about autism and ESDM strategies among the preschool staff.

#### 3.4.2 Positive impact but still a need for intervention

The parents experienced that the intervention had had a positive impact on the child's development in several areas, such as in the core symptoms of autism, adaptive behaviour as well as reduced prevalence of behaviour problems. “*Now [the child]is open to other children and people/…/you could say that the package is open*” (9). Additionally, the child's improvement contributed to a sense of well-being for the parents. “*Then the results started showing, then you started getting more motivation, you started to become happier*” (12). The parents underscored that the intervention yielded benefits, even if the child still needed continued support in different areas.

#### 3.4.3 Improved collaboration

The shared objective-setting and regular network meetings were central in creating enhanced collaboration between healthcare, families, and preschool. The goal setting created a shared view of the child's development and what the child needed, and the child received the opportunity to practice the skills at home and in preschool.

The network meetings also allowed parents to talk about the child's development with the preschool staff. In the daily interactions, it was hard to find time to talk with the preschool staff about the child, and sometimes, it was sensitive to discuss the child's difficulties in the presence of other parents. “*Also, I don’t want to talk about my daughter's private issues when another parent is standing there*” (12). The network meetings also contributed to solving challenges between the parents and preschool staff. During the network meeting, different views could be discussed, and the ESDM therapist was described by the parents as supportive in these situations.

## 4 Discussion

A compelling body of research supports the need for early detection and intervention for children with autism (10, 11). Still, several studies have shown that there are barriers in accessing equal healthcare and support in migrant populations, often resulting in delayed or missed autism detection, diagnosis, and interventions (8, 30). This study aimed to describe how parents to children with autism experienced participating in the intervention program based on ESDM.

In the present study, the parents believed that *the intervention program's goals were appropriate*. As previously reported, the parents underscored the comprehensive approach of the intervention program, including early detection and intervention in their local setting, individualized goals based on their child's needs and prioritization, collaboration, and intervention targeting different developmental areas as essential (15).

It was shown in a previous study by Linnsand et al. (35) that the multidisciplinary team's local availability was essential when it came to enhance the accessibility and continuity of healthcare services. Families participating in our study were enrolled from the local community and came from diverse geographical and educational backgrounds. The setting of individualized goals and strategies for each participating child and their family were pivotal to meeting these different needs, this have also been highlighted in earlier studies (33, 40).

In addition to the parent's needs, the children in the study also needed support in different areas. In general, children with autism are at high risk of having comorbid neurodevelopmental disorders and medical conditions (41–45), and several studies have found a pattern of more significant intellectual impairments and more severe autism symptoms among children of parents with migrant background (46–48). Despite the autism core symptoms, the children in the present study had several related difficulties. The ESDM intervention targets autism-specific impairments spanning over various developmental domains, which made the intervention suitable for the included children, that may have increased the social validity in the views of the parents.

The comprehensive approach in the intervention described in this study seemed to contribute to the significant goals of the intervention program as perceived by the parents. The goals of the intervention also matches well with the recommendations for interventions for young children with autism that also was reported previously by Zwaigenbaum et al. (11), such as integrating developmental and behavioural approaches, active involvement of caregivers, focus on autism core symptoms and related difficulties.

Previous research on ESDM has evaluated the intervention's efficacy (13). In the present study, the parents highlighted some central aspects of the ESDM procedure as being beneficial and helpful, including the focus on the child's interest and motivation, shared objective-setting, regular network meetings, and the possibility of applying the goals in daily routines. Also, in a study by Ogilvie and McCrudden (27), the parents described the ESDM procedure as child orientated. The parents in our study highlighted, similarly previous studies, the naturalistic procedures and how such strategies could easily be incorporated into already established routines in different settings (27, 35).

The multidisciplinary team's local attachment was perceived as a pivotal part of the procedure to enhance accessibility by the parents. Also, in other studies on people with migrant background, local access to treatment has been highlighted as important; geographic barriers make seeking care more difficult (49, 50). Not just the local accessibility was important for the parents in this study, also, the ESDM therapist emotional availability and helpful attitude was described as essential. Both the children and their parents had multiple needs, which meant that the need for both practical and emotional support. The parents needed information and support to understand and navigate the Swedish public health and social services systems. This result is in line with other studies from migrant communities (49, 51), but in a study conducted in the same city as the current study, non-migrant parents also perceived navigating the public system as challenging (52). So, difficulties in navigating the healthcare system in Sweden may be a general issue, and more research is needed in that field.

One of the subthemes in this study was about gaining knowledge about autism, and the parents received knowledge during the parent education and the network meetings. During the network meeting, the ESDM therapist coached the parents and preschool staff in implementing and achieving the objectives for each specific child into the daily activities. Also, other studies have highlighted the importance of education about autism, especially for people with migrant background, where knowledge of autism often is limited (49). Thereby, this study may have contributed to a change in that aspect.

In social validity, the outcome must be evaluated on whether the intervention effect is significant to the participants. The theme in this study describing social importance is named “A sense of empowerment”, and the parents experienced that *the intervention program's outcome was significant* within the family's daily living. It is a critical outcome as the parents primarily influence their children's learning and development in the home environment. Involving parents in delivering interventions to children with autism may improve their ability to support learning and development in their children and their capacity to practice and generalize the intervention across natural settings (18, 53). We believe that enhancing parents' capacity to support their children and strengthening their confidence in that they can help their child is essential. Thereby, this intervention reinforces the parent's role as vital in the child's development, even after the intervention is ended.

An essential aspect of social importance was probably the fact that the parents experienced that the intervention positively impacted their child's development. Improved collaboration with the preschool staff was also a significant outcome of the intervention program described by the parents in the present study. The parents, as well as the preschool staff within the study by Linnsand et al. (35), emphasized that the shared objectives and network meetings were central to promoting enhanced collaboration. As shown with in our result, another benefit of the intervention program contributed to an improved knowledge about autism and ESDM strategies among the parents as well as the preschool staff. The parents were educated about autism and the techniques for fostering avenues of communication and interaction with the child within daily routines. Based on this, delivering a high-quality intervention is dependent on a good general knowledge of autism, as well as competence in the intervention model, among all that are involved in delivering the intervention.

The intervention's social validity was essential during the intervention period and afterwards, as the parents may impact the possibility of maintaining the strategies. In our material, the parents experienced that the intervention positively impacted the child's development, but the child needed continued support in different areas. Therefore, it is crucial that the parents can use and, if required, develop the strategies learned in the intervention after the formal support of use is ended. The maintenance and implementation of what the parents learned from the intervention described in this study would be interesting to study in future research. A growing body of research attest to the feasibility and acceptability of implementing evidence-supported interventions within community settings (17, 27, 54). The maintenance and implementation of what the parents learned in this study may be increased as the intervention was performed within community settings.

### 4.1 Methodological considerations: strengths and limitations

Methodological aspects of trustworthiness within our study were considered in terms of credibility, dependability, confirmability and transferability (55). A special guide was used for the interviews to ensure that the questions stayed close to the aim of the study which strengthen dependability. Using quotes from the interviews to illustrate subthemes ensured confirmability, and reflexivity, as described by Polit and Beck (56) was undertaken when continuous and recurrent discussions between authors were held during the analysis.

In qualitative research, a heterogeneity among participants is important if one wants to be able to capture as broad and deep representation as possible of parents' experiences. Within our study parents with different backgrounds were included, including age, education, language background, and sex. This could be considered as a strength and support the credibility of the findings. Additionally, data collection stopped when saturation was reached. The description of the included participants and researchers strengthen the transferability (37).

### 4.2 Methodological limitations

The study context is within a specific area that had a high prevalence of immigrants which could have an impact on the generalizability of the results, however, the sample and the area are described thoroughly to give the reader sufficient information when to interpretate the results. A part of our aim was to also include participants that often are excluded in other interview studies, those who do not speak the major language.

Nearly 50% of the interviews were performed with an authorized interpreter, which could be counted as both strength and limitation. Our intention was to capture as many different attitudes as possible and to give parents with different backgrounds the possibility to be included, thus involving interpreters was essential. However, the limitation is that the language does not become as natural and fluid as if you speak directly with a parent without using an interpreter. We think that the risk of this influencing the results was low since experienced interviewer and authorized interpreters were involved. When uncertainties arose, the interviewer asked for clarification, which was used more frequently to ensure that the parents conveyed their message clearly.

The findings in this study included mostly positive experiences, there could be a risk that the parents were influenced by the personal connection and contact with the clinic, thus the interviewer was not involved with the intervention program which may have decreased the potential bias. The researchers brought different perspectives into the analysis, that may have improved the quality of the analysis.

### 4.3 Future research

Our study focuses on parents' perspectives of an intervention based on ESDM. In a future study, it would be interesting and valuable to include the children's progress, as well as a measure of social validity through a questionnaire.

## 5 Conclusions

To summarize, the parents believed that the naturalistic, comprehensive and local intervention, led to gained knowledge about autism and a sense of empowerment to handle the child's autism symptoms. The experiences from the parents' showed that the intervention goals were perceived as appropriate for their children, had acceptable procedures, and produced meaningful outcomes. The results indicated that the intervention program based on the ESDM was socially valid according to the parents' descriptions.

## Data Availability

The datasets presented in this article are not readily available because data is not anonymized. Therefore, access to data can be granted after an approved application to The Ethical board Sweden and other internal legal procedures authorize external access to the data. Requests to access the datasets should be directed to petra.linnsand@vgregion.se.
